# Fatal Human Infection with *Rickettsia rickettsii*, Yucatán, Mexico

**DOI:** 10.3201/eid1204.051282

**Published:** 2006-04

**Authors:** Jorge E. Zavala-Castro, Jorge E. Zavala-Velázquez, David H. Walker, Edgar E. Ruiz Arcila, Hugo Laviada-Molina, Juan P. Olano, José A. Ruiz-Sosa, Melissa A. Small, Karla R. Dzul-Rosado

**Affiliations:** * Universidad Autónoma de Yucatán, Mérida Yucatán, México;; † University of Texas Medical Branch at Galveston, Galveston, Texas, USA;; ‡ Hospital General Agustín O'Horán, Merida, Mexico

**Keywords:** Rocky Mountain spotted fever, Rickettsia rickettsii, immunohistochemistry, molecular diagnostic techniques

## Abstract

The first fatal *Rickettsia rickettsii* infection was diagnosed in the southwest of Mexico. The patient had fever, erythematous rash, abdominal pain, and severe central nervous system involvement with convulsive crisis. The diagnosis of *R. rickettsii* infection was established by immunohistochemistry and specific polymerase chain reaction.

Five spotted fever group (SFG) rickettsioses have been documented in the Western Hemisphere: Rocky Mountain spotted fever (RMSF) (*Rickettsia rickettsii*), fleaborne spotted fever (*R. felis*), rickettsialpox (*R. akari*), African tick-bite fever (*R. africae*), and infection with *R. parkeri*. *R. rickettsii* infections have been identified in southern Canada, the United States, northern Mexico, Costa Rica, Panama, Brazil, and Argentina (1–5). *R. felis* has been detected in humans, *Ctenocephalides* fleas, and opossums in the United States, Mexico, Brazil, Uruguay, and Peru (6–9); *R. parkeri* in the United States, Uruguay, and Brazil (10); *R. africae* on islands of the Caribbean Sea; and *R. akari* in the United States. Among these agents, only *R. rickettsii* is known to cause fatal infections. The only SFG rickettsial agent previously documented to cause human infections in the Yucatán Peninsula of Mexico, where >5% of the population have antibodies to SFG rickettsiae, is *R. felis*, which is present in 20% of cat fleas (*Ctenocephalides felis*) (7,11). Previously, Rocky Mountain spotted fever had been recognized mainly in northern Mexico, beginning in the 1940s (Figure 1). We report a case of fatal RMSF in a previously healthy child in southwestern Mexico, where this infection had not previously been recognized. The case may herald the reemergence of RMSF throughout the Americas or be evidence of a misdiagnosed disease in Latin America.

**Figure 1:**
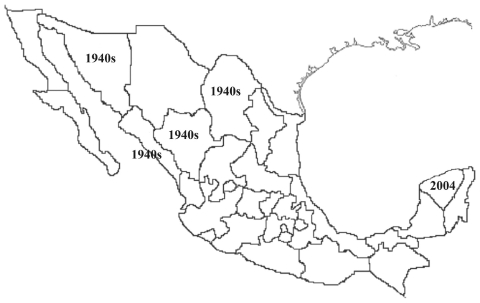
Map of Mexico showing the period and regions where human cases caused by *Rickettsia rickettsii* were detected.

## The Case

In August 2004, a previously healthy girl, age 4 years and 9 months, was found with 2 ticks attached to her left ear lobe 3 days before the onset of fever and headache. She was treated with amoxicillin and had a progressively severe illness with fever, abdominal pain, headache, fatigue, diarrhea, nausea, vomiting, cutaneous paresthesias, myalgia, rigidity of the left arm and both legs, and an erythematous rash involving the extremities and thorax. At the site of tick attachment on the left ear, an eschar was observed in association with tender regional lymphadenopathy at the time of admission. Clinical laboratory evaluation showed elevated serum urea and hepatic transaminase concentrations and neutrophil leukocytosis. Thrombocytopenia was not reported. On day 7 of illness, seizures developed, and the patient died.

**Figure 2:**
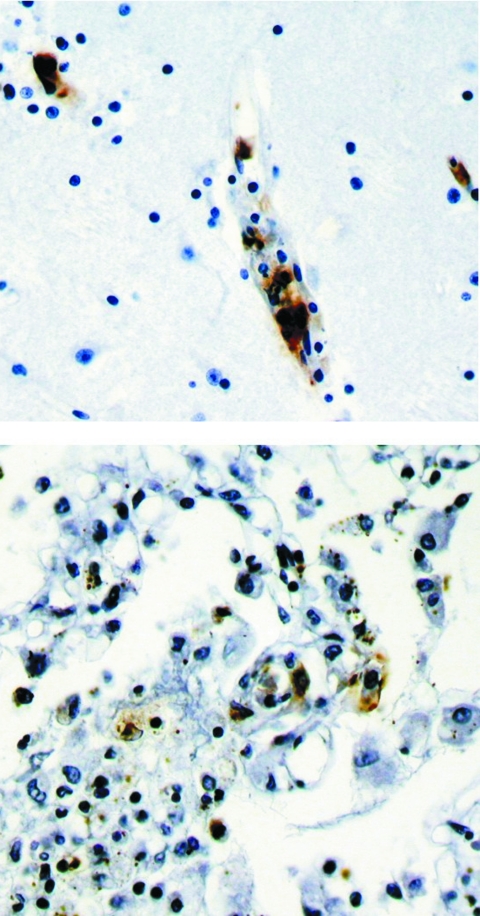
Immunohistochemical stain shows spotted fever group rickettsia in endothelial cells of a blood vessel in brain (top panel) and lung (bottom panel).

All research was approved by the ethics committee of the Faculty of Medicine, Universidad Autonoma de Yucatán. Necropsy showed cerebral edema and hemorrhages in the pleura, lungs, pericardium, endocardium, and gastric mucosa. Histopathologic examination demonstrated many lesions of lymphohistiocytic vasculitis, characteristic of rickettsial infection. Immunohistochemical staining performed with specific monoclonal antibodies against SFG lipopolysaccharide as described previously (4) identified SFG rickettsiae in vascular endothelial cells in multiple foci in the brain, lung, spleen, and liver (Figure 2).

The patient lived in an urban area where many dogs and sheep also lived. Two months before the onset of the patient's illness, seizures developed in a dog and a sheep belonging to the family; both died.

DNA was extracted from paraffin-embedded spleen and formaldehyde-fixed liver, lung, and brain tissue by DNeasy Tissue kit (Qiagen, Valencia, CA, USA) as previously described. Polymerase chain reaction (PCR) amplification of the extracted DNA used genus-specific primers for the rickettsial 17-kDA protein gene, 5´-TGTCTATCAATTCACAACTTGCC-3´ and 5´-GCTTACAAAATTCTAAAAACCATATA-3´. The fragment was cloned into the TPO TA pCR 2·1-TOPO vector (Invitrogen, Frederick, MD, USA), and selected clones from the same cloning reaction were sequenced 3 times with a ABI Prism 377 automated sequencer (Perkin Elmer, Foster City, CA, USA), and the sequences were compared to those in the GenBank database by using the Basic Local Alignment Search Tool at the National Center for Biotechnology Information (12). Two clones (GenBank accession no. DQ176856) identified the DNA sequence of the 434-bp product as *R. rickettsii* (GenBank accession no. AY281069), which differed by only 1 nucleotide.

## Conclusions

The first documentation of RMSF in southwestern Mexico reflects, in part, the development of a regional research laboratory with knowledge and interest in rickettsiology, a situation that is lacking in most parts of Latin America. However, this finding may also represent an early warning of widespread reemergence of RMSF. In the United States, 2 large waves of emergence of RMSF have been documented during the last century; peaks were seen in the mid-1940s and early 1980s. In 2004, a total of 1,514 cases of RMSF were reported, the highest number ever in a single year, including an outbreak in Arizona, where very few cases had been diagnosed previously (13).

The recent diagnosis of the first cases of RMSF in Argentina (5), reemergence of RMSF in large clusters with a case-fatality ratio of 50% in Brazil (4), and reemergence of isolation-documented fatal RMSF in Colombia suggest that the factors responsible for the increased incidence are widespread. This phenomenon was noted for the parallel reemergence of RMSF and Mediterranean spotted fever during the 1970s and 1980s (14). The ecologic and epidemiologic factors responsible for the periodically increased transmission of *R. rickettsii* from ticks to humans have not been determined.

Most aspects of this fatal case are typical of RMSF: tick bite, illness in a dog at the residence, disseminated lymphohistiocytic vasculitis, acute renal failure, and fatal seizures associated with cerebral rickettsial endothelial infection, increased vascular permeability, and cerebral edema. However, other features are unusual for RMSF. Although eschars are common in most SFG rickettsioses, they have seldom been documented in RMSF (15). Despite the hypothetical spread of SFG rickettsiae from the site of tick feeding through lymphatic vessels to regional lymph nodes, regional lymphadenopathy is not a typical feature of RMSF. Moreover, hemorrhages are not a prominent feature in most cases of RMSF in North America, compared with reports of severe hemorrhages in cases from Brazil. Whether such clinical and pathologic differences are real or not remains to be determined as well as their potential association with genetic differences in rickettsial virulence factors or host factors, including deleterious effects of medications taken early in the course of illness.

This case illustrates the major deficiency in controlling RMSF, the lack of a diagnostic test that is effective early in the course and widely available. Patients seldom have antibodies to *R. rickettsii* when they are first seen by a clinician. PCR detection of rickettsial DNA in blood is insensitive, particularly early in the course. Diagnostic immunohistochemistry and PCR are available in only a few reference laboratories. Timely consideration of the diagnosis and empiric treatment with doxycycline are the best that can be achieved in most settings in Mexico, the United States, or elsewhere.
